# Supplementary figures and images for: Time course analysis of large-scale gene expression in incised muscle using correspondence analysis
Source: PLoS One. 2020 Mar 25;15(3):e0230737. doi: 10.1371/journal.pone.0230737 (PMC7094855; doi:10.1371/journal.pone.0230737)

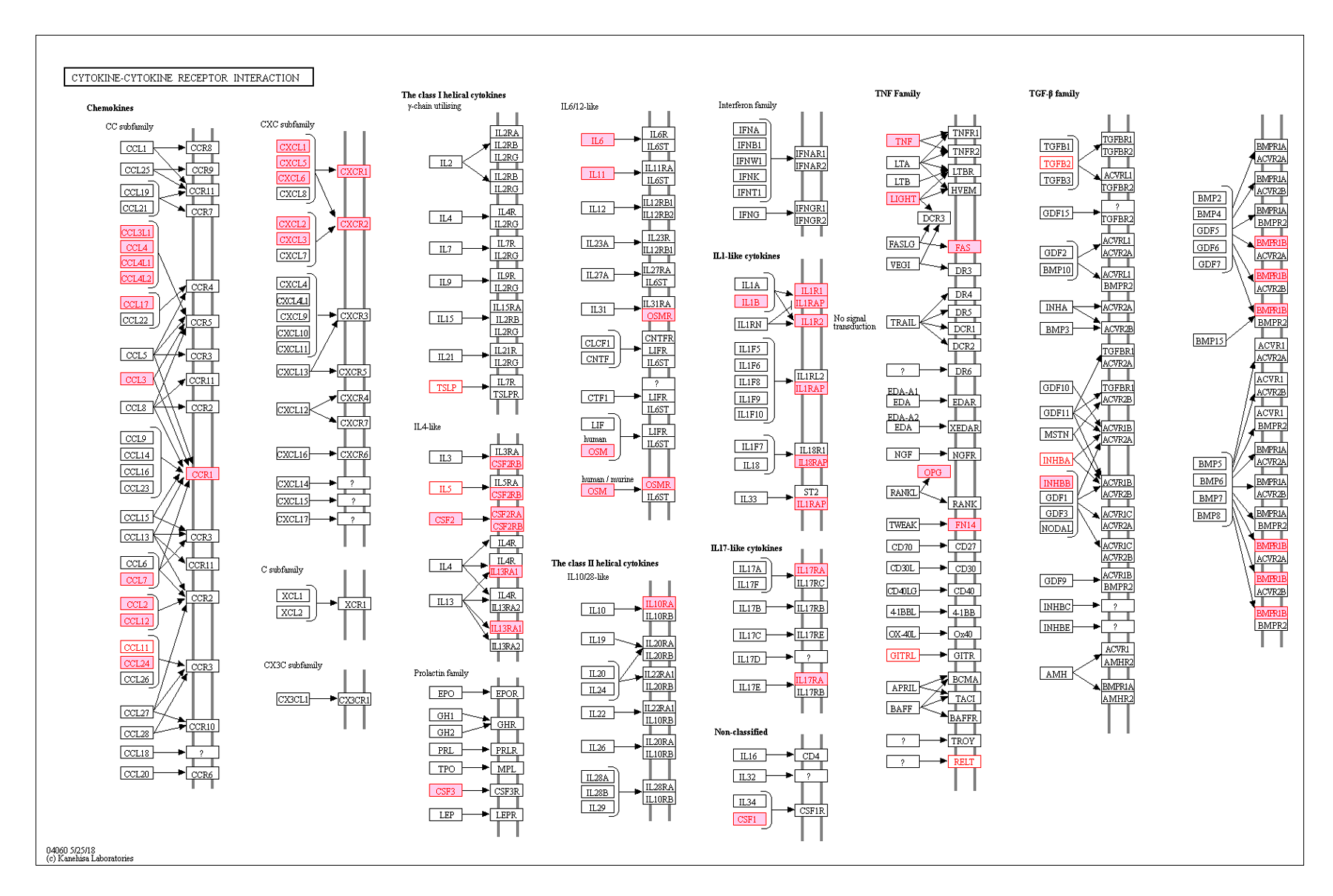

Supplement: S1 Fig — This pathway was also significant in the top 1,000 gene set whose distances are close to Cxcl5 on the CA plot. In the Cxcl5 gene set, the FC values of many genes belonging to this pathway are more than 1 throughout time course (shown in red filled frame), but FC24 of some genes is less than 1 (red frame). This is because Cxcl5 was located near the vertex of tAg where genes with large FC values tended to gather, and this set consisted of genes distributed in the area of C and D in Fig 6 (FC24 was the smallest at all times). The figure was created with KEGG Mapper (https://www.genome.jp/kegg/mapper.html). 10.6084/m9.figshare.10327334. (TIF) [file pone.0230737.s001.tif]

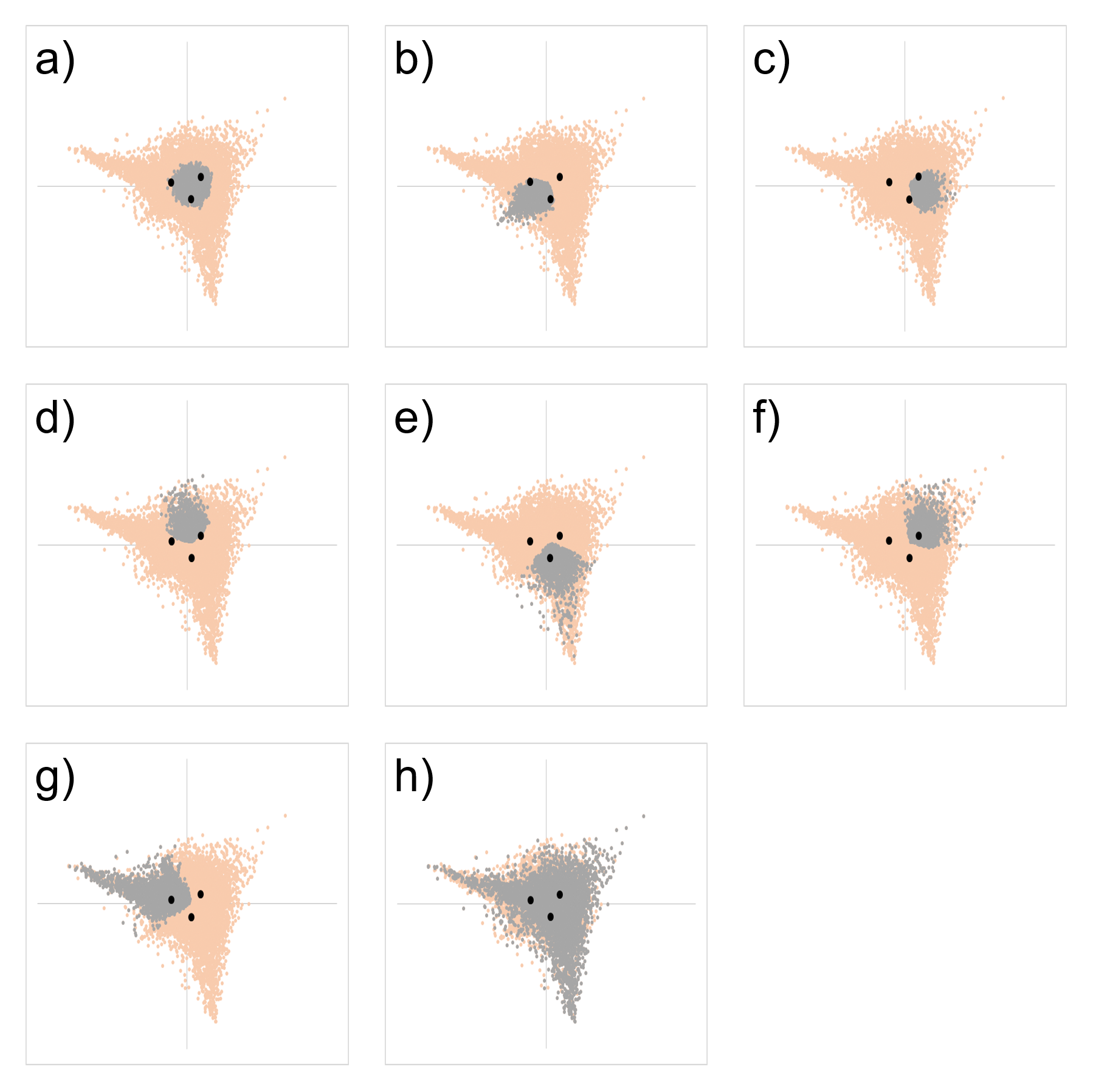

Supplement: S2 Fig — Genes corresponding to the following conditions are shown in gray on the CA plot. a): FC6, FC12, FC24 < 1; b): FC12, FC24 < 1 < FC6; c): FC6, FC24 < 1 < FC12; d): FC6, FC12 < 1 < FC24; e): FC24 < 1 < FC6, FC12; f): FC6 < 1 < FC12, FC24; g): FC12 < 1 < FC6, FC24; h): 1 < FC6, FC12, FC24. Genes formed fairly clear clusters on the plot according to whether each of the 3 FC values was greater than 1. Genes that did not satisfy each condition and the 3 time series scores are shown in orange and black dots, respectively. (TIF) [file pone.0230737.s002.tif]

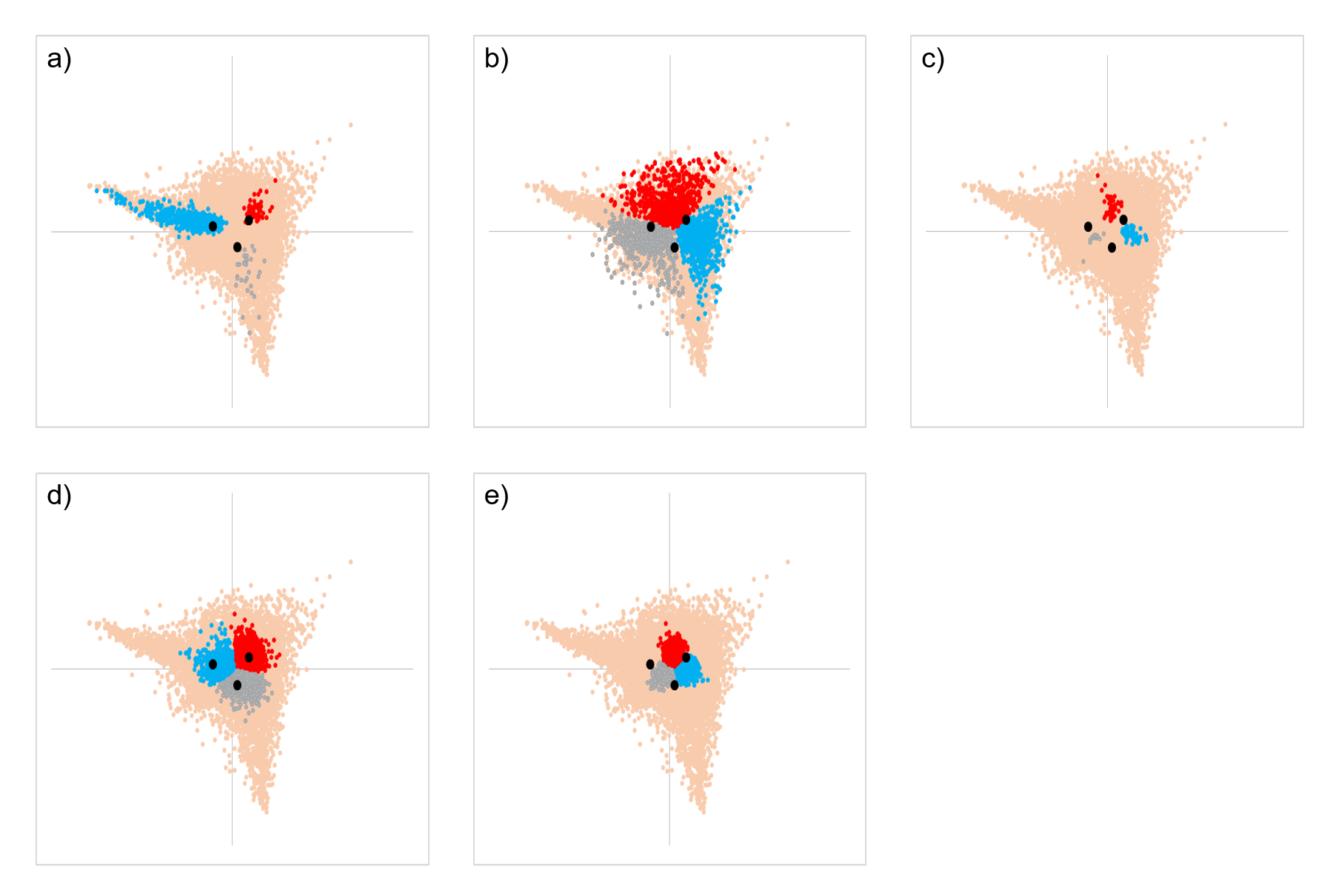

Supplement: S3 Fig — Plot of genes that have two time series significantly upregulated and one time series significant downregulated. Gray dots: (U, U, D); blue dots: (U, D, U); red dots: (D, U, U). b): Plot of genes that have one time series significantly upregulated and two time series without fluctuation. Gray dots: (U, -, -); blue dots: (-, U, -); red dots: (-, -, U). c): Plot of genes that have one time series significantly upregulated and two time series significantly downregulated. Gray dots: (U, D, D); blue dots: (D, U, D); red dots: (D, D, U). d): Plot of genes that have one time series significantly downregulated and two time series without fluctuation. Gray dots: (-, -, D); blue dots: (-, D, -); red dots: (D, -, -). e): Plot of genes that have two time series significantly downregulated and one time series without fluctuation. Gray dots: (-, D, D); blue dots: (D, -, D); red dots: (D, D, -). Genes with the same fluctuation direction are plotted at symmetrical positions with each other across the center of tAg according to the combination of fluctuation behavior at each time point. Genes that did not satisfy any condition and the 3 time series scores are shown in orange and black dots, respectively. (TIF) [file pone.0230737.s003.tif]

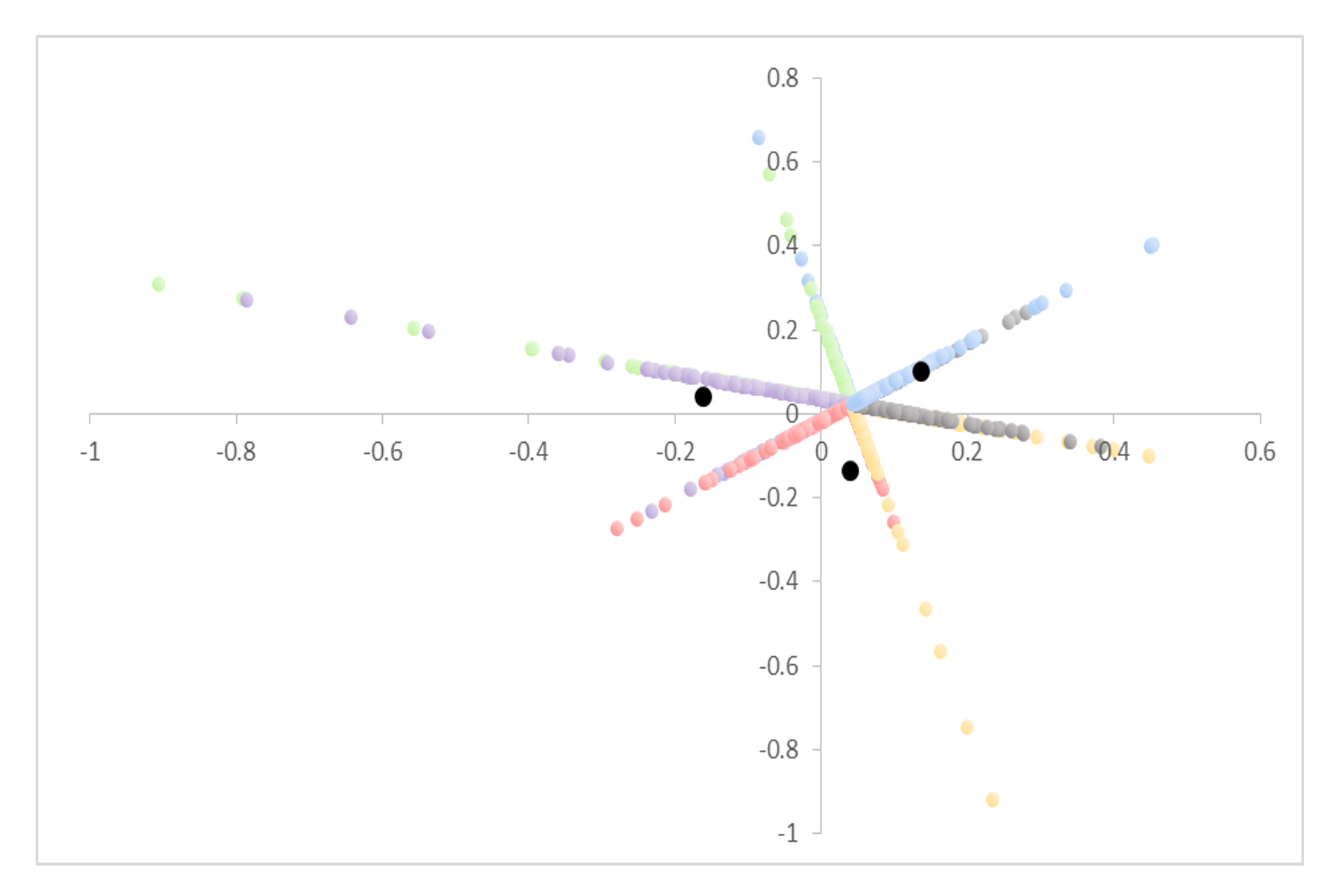

Supplement: S4 Fig — For each gene, (highest FC value among the three time points)/(middle FC value) and (middle FC value)/(lowest FC value) were calculated, and the top 100 genes whose ratio was close to 1 were selected and plotted. Blue dots: FC6/FC12 and FC12/FC24 were close to 1 (area A in Fig 5). Gray dots: FC24/FC6 and FC24/FC12 (area B). Yellow dots: FC24/FC6 and FC12/FC6 (area C). Red dots: FC24/FC12 and FC12/FC6 (area D). Purple dots: FC24/FC12 and FC24/FC6 (area E). Green dots: FC12/FC6 and FC24/FC6 (area F). In each area, selected genes were distributed along the two border lines. Black dots indicate 3 column scores. (TIF) [file pone.0230737.s004.tif]

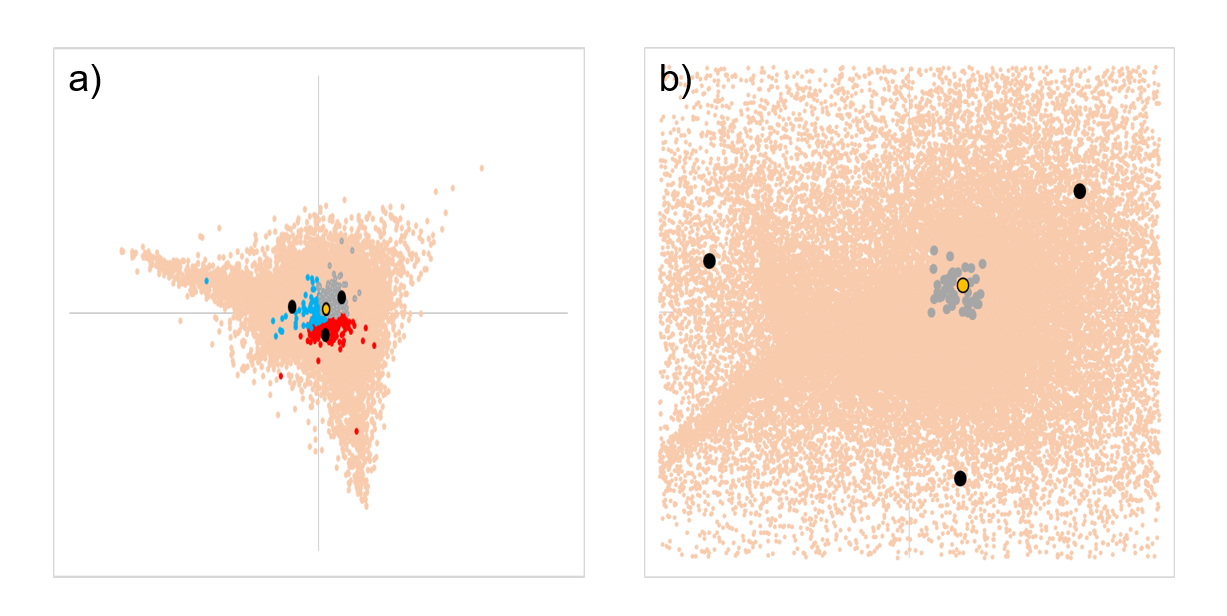

Supplement: S5 Fig — Yellow dot indicates the approximate intersection of the six areas in Fig 6 on the CA plot (point P). a): Plot of genes with FC values less than 0.2 at a specific time point. Gray dots: 6 h; blue dots: 12 h; red dots: 24 h. b): Magnified image around point P; the 46 genes with FC values less than 0.2 at all time points are shown in gray dots. There was a tendency for genes with small FC values to gather around point P. Genes that did not satisfy any condition and the 3 time series scores are shown in orange and black dots, respectively. (TIF) [file pone.0230737.s005.tif]

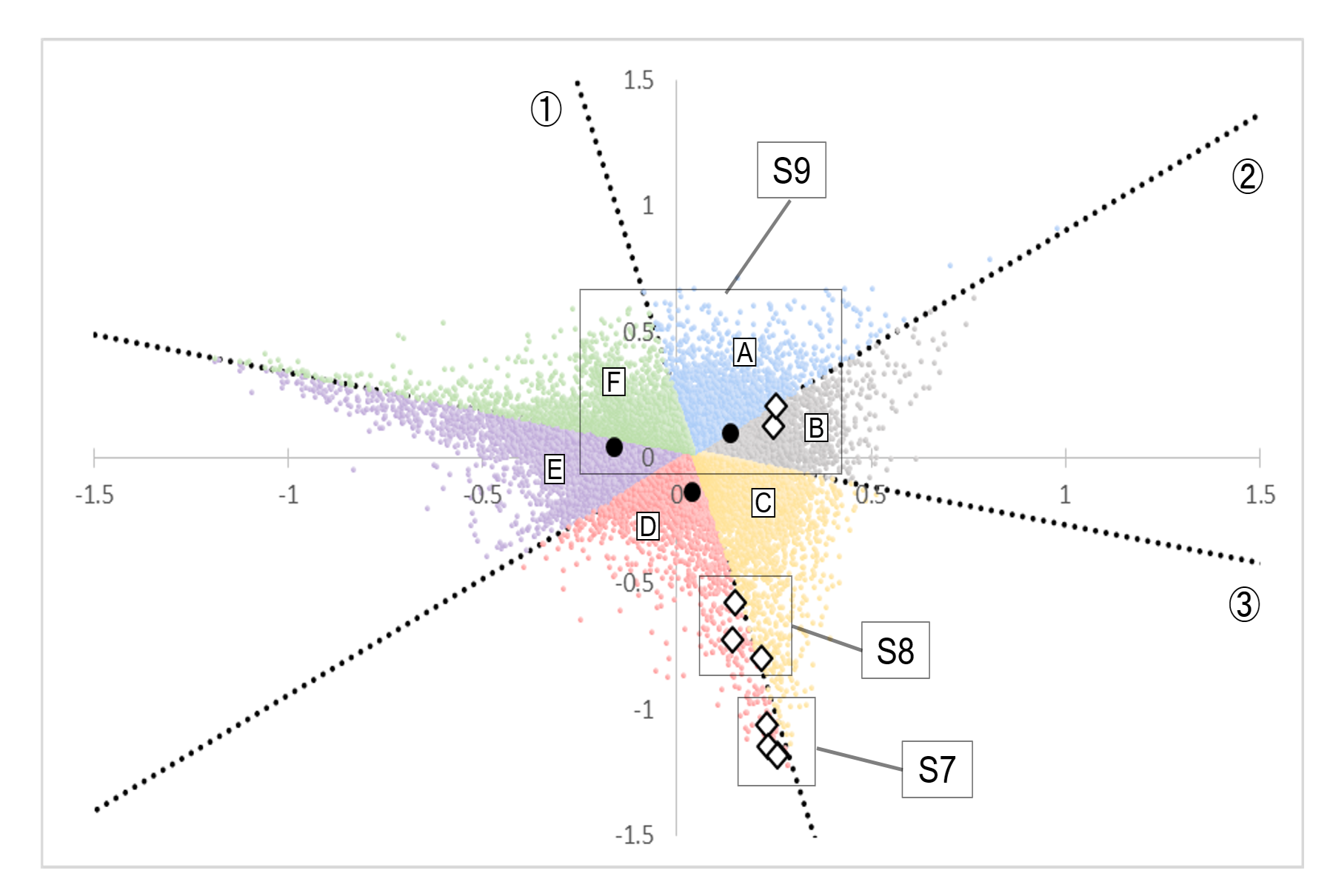

Supplement: S6 Fig — Magnified images around each upregulated query gene are shown in S7, S8 and S9 Figs. Black dots indicate the 3 time series scores. (TIF) [file pone.0230737.s006.tif]

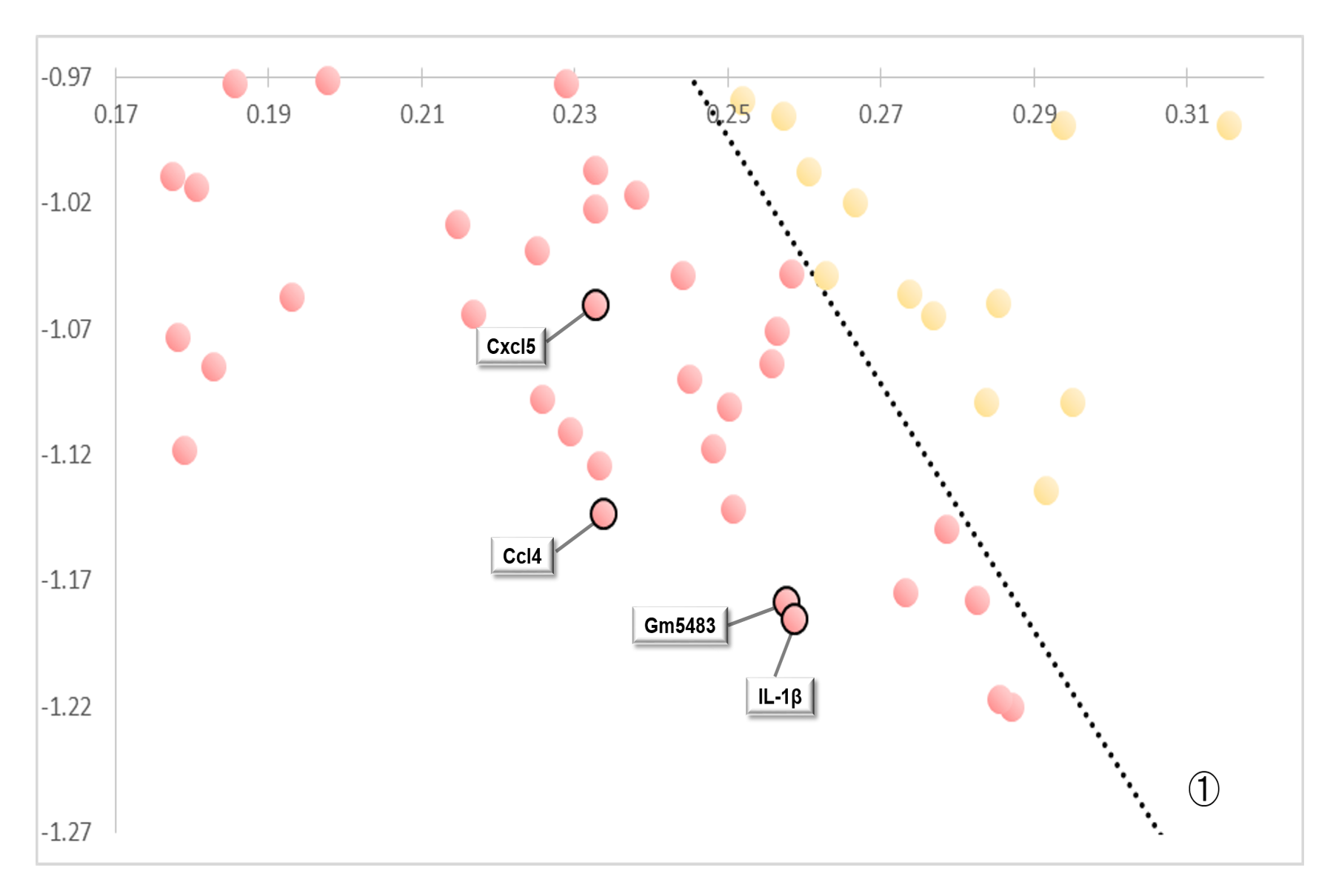

Supplement: S7 Fig — Other genes in the area of C and D, and straight line 1 are also shown. (TIF) [file pone.0230737.s007.tif]

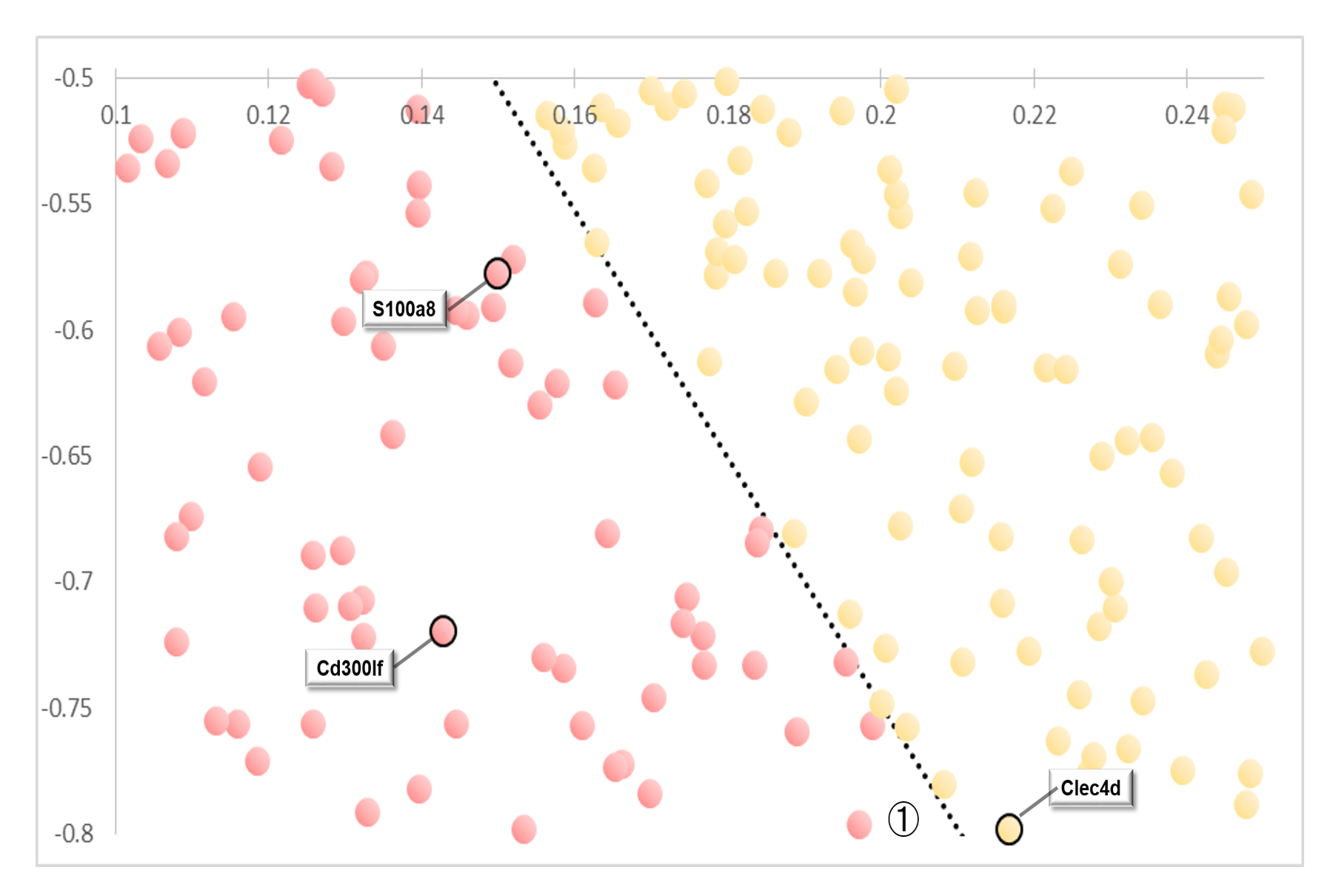

Supplement: S8 Fig — Other genes in the area of C and D, and straight line 1 are also shown. (TIF) [file pone.0230737.s008.tif]

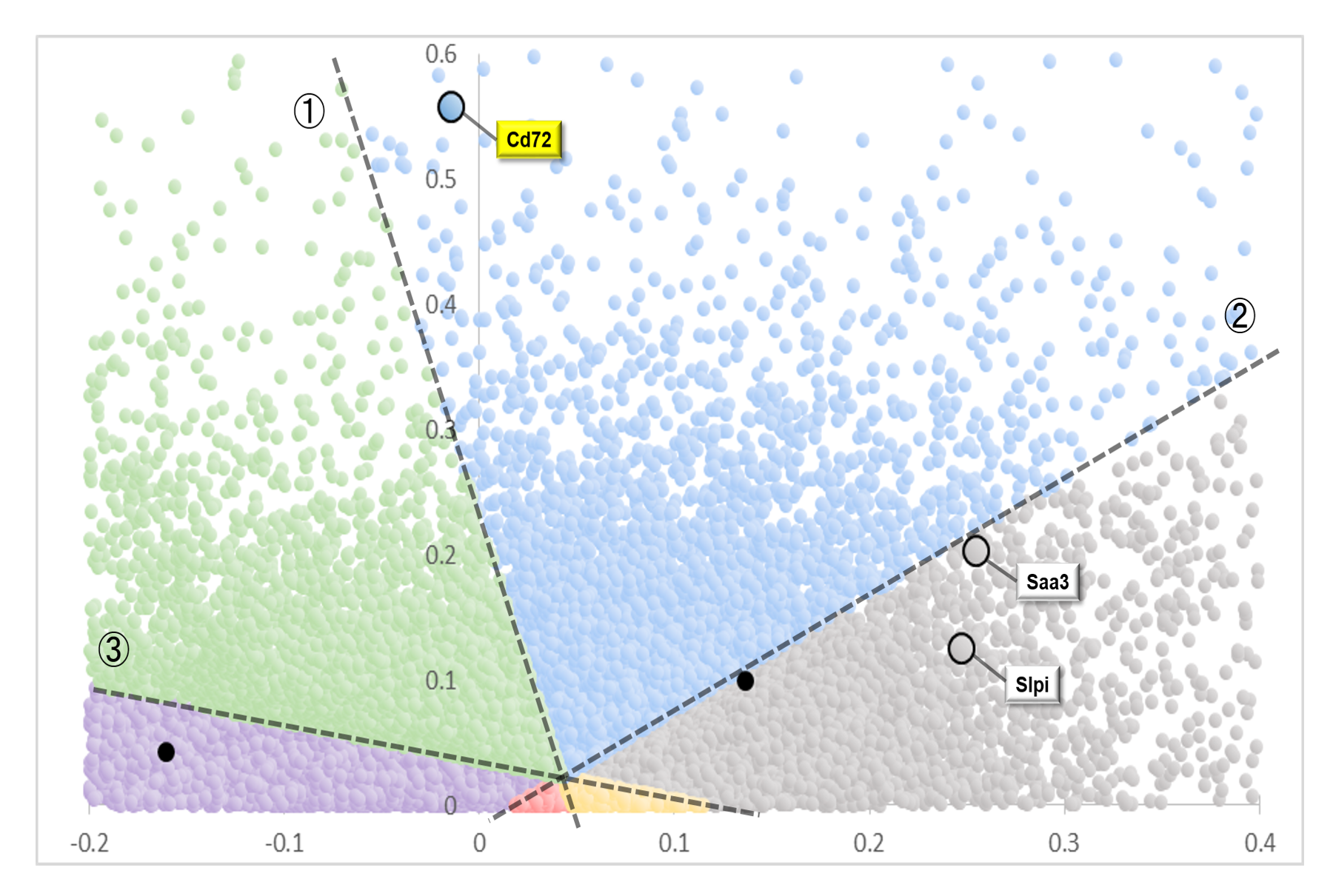

Supplement: S9 Fig — Cd72 is shown for comparison with the other upregulated query genes. The 2 time series scores (black dots), other genes, and three straight lines are also shown in this figure. (TIF) [file pone.0230737.s009.tif]
