# Supplementary material for: Time course analysis of large-scale gene expression in incised muscle using correspondence analysis
Source: PLoS One. 2020 Mar 25;15(3):e0230737. doi: 10.1371/journal.pone.0230737 (PMC7094855; doi:10.1371/journal.pone.0230737)
Supplement: S1 Appendix — (DOCX) [file pone.0230737.s011.docx]

*Correspondence analysis*

On the plot, the magnitude relationship between FC6 and FC12 was reversed with the approximate straight line 1 as the border, and so was FC12 and FC24 with line 2, and FC24 and FC6 the line 3. The time series scores of 6, 12, and 24 h were located in the areas of B (near the boundary with the area A, E, and D respectively. Therefore, the 3 lines were not identical with the straight lines passed through each time score and origin of the coordinate (0, 0); instead, they would have intersected at point P or coordinate (0.04266, 0.02246) based on the calculation. Moreover, there was a tendency for genes whose FC values were almost equal throughout time, namely genes that were upregulated, downregulated, or did fluctuate throughout time, to be distributed in the neighborhood of P (Fig. 5e). In contrast, we expected that the ratio of FC values of each gene should have increased as the distance from the straight lines increased. For example, the value of FC12/FC6 of the genes in the area A of Fig. 6 was expected to increase as the distance from the line 1 increased, although they did not necessarily arrange in a perfect ascending order (S1 Table A). We also expected that the variance of all 3 FC values of the genes would become larger as their location was farther from P, although again not in perfect ascending order (S2 Table B).

**S1 Table A. The value of FC12/FC6 and the distance between each gene and straight line 1 in Fig. 6.**

| Probe name | FC12/FC6 | Distance |
| --- | --- | --- |
| A_55_P2027436 | 1.00001 | 1.10943E-17 |
| A_52_P379277 | 1.00020 | 1.44644E-05 |
| A_51_P148675 | 1.00017 | 2.29589E-05 |
| A_52_P450835 | 1.00023 | 3.57905E-05 |
| A_55_P2177968 | 1.00021 | 4.46634E-05 |
| A_55_P2145109 | 1.00035 | 9.73649E-05 |
| A_55_P2047842 | 1.00114 | 1.07540E-04 |
| A_51_P364657 | 1.00195 | 1.35290E-04 |
| A_51_P391926 | 1.00100 | 1.86956E-04 |
| … | … | … |
| A_30_P01018914 | 3.12942 | 0.55440 |
| A_55_P1988975 | 3.48187 | 0.55510 |
| A_51_P196695 | 3.46452 | 0.56859 |
| A_51_P136294 | 3.15514 | 0.58026 |
| A_51_P484054 | 4.83767 | 0.59020 |
| A_55_P2081432 | 3.57978 | 0.59334 |
| A_51_P400543 | 4.68617 | 0.63512 |
| A_55_P2004007 | 6.76196 | 0.79601 |
| A_51_P426195 | 8.81806 | 0.90079 |
| A_51_P303160 | 22.82841 | 1.09659 |

"The table shows 10 genes each in the upper and lower order when arranged in ascending order by distance value."

**S1 Table B. The variance of the FC value at all time points and the distance between the gene and point P in Fig. 6.**

| Probe name | variance of FC value | Distance |
| --- | --- | --- |
| A_55_P2361547 | 1.01620E-05 | 0.00154 |
| A_55_P2138257 | 6.50663E-06 | 0.00166 |
| A_30_P01028331 | 6.05139E-06 | 0.00170 |
| A_55_P2044385 | 5.55952E-06 | 0.00174 |
| A_55_P2005813 | 7.16589E-06 | 0.00194 |
| A_30_P01031914 | 1.16328E-05 | 0.00229 |
| A_30_P01020724 | 2.18136E-05 | 0.00339 |
| A_55_P2126469 | 2.72966E-05 | 0.00351 |
| A_51_P123297 | 3.35989E-05 | 0.00415 |
| A_55_P2108768 | 4.95278E-05 | 0.00496 |
| … | … | … |
| A_51_P223709 | 7.42886 | 0.71670 |
| A_55_P2020538 | 22.16657 | 0.72593 |
| A_30_P01018914 | 93.56448 | 0.72667 |
| A_55_P2081432 | 61.51453 | 0.73477 |
| A_51_P400543 | 6.71696 | 0.75524 |
| A_55_P2035286 | 11.66853 | 0.75581 |
| A_51_P136294 | 270.32928 | 0.79577 |
| A_55_P2004007 | 38.99594 | 0.99341 |
| A_51_P426195 | 110.94287 | 1.08154 |
| A_51_P303160 | 6818.09196 | 1.29243 |

"The table shows 10 genes each in the upper and lower order when arranged in ascending order by distance value."
