# Supplementary material for: Time course analysis of large-scale gene expression in incised muscle using correspondence analysis
Source: PLoS One. 2020 Mar 25;15(3):e0230737. doi: 10.1371/journal.pone.0230737 (PMC7094855; doi:10.1371/journal.pone.0230737)
Supplement: S2 Appendix — (DOCX) [file pone.0230737.s012.docx]

*Analyses of genes close to query gene scores (distance 2)*

Among the upregulated query genes, *Cxcl5*, *Gm5483*, *Ccl4*, and *Il-1β* were located close to each other near the lower vertex of ΔAg, resulting in their top 100 gene sets being all identical. Similarly, 298 out of the top 300, and 995 out of the top 1,000 gene sets were shared by the 4 query genes. Accordingly, the significant GO terms were almost identical, and the significant pathways were completely matched among the gene sets (S2 Table A). On the other hand, *Slpi* and *Saa3* distributed near the coordinate score for 6 h shared 0, 59, and 520 genes in their top 100, 300, and 1,000 sets, respectively. The distance between *Slpi* and *Saa3* was 0.078, which was smaller than that between *Cxcl5* and the other 3 genes (*Gm5483*, *Ccl4*, and *Il-1β*, 0.083 to 0.128). The reason why there were fewer shared genes in *Slpi* and *Saa3* despite the comparable distance was that genes without fluctuation were concentrated in neighborhood of the center of ΔAg (Fig. 7d).

**S2 Table A. Significant pathways in the analysis of the top 1000 gene set of *Cxcl5*.**

| Entry | Name |
| --- | --- |
| mmu05143 | African trypanosomiasis |
| mmu04933 | AGE-RAGE signaling pathway in diabetic complications |
| mmu05146 | Amoebiasis |
| mmu05142 | Chagas disease (American trypanosomiasis |
| mmu04610 | Complement and coagulation cascades |
| mmu04060 | Cytokine-cytokine receptor interaction |
| mmu05418 | Fluid shear stress and atherosclerosis |
| mmu04640 | Hematopoietic cell lineage |
| mmu04657 | Il-17 signaling pathway |
| mmu05321 | Inflammatory bowel disease (IBD |
| mmu04630 | Jak-STAT signaling pathway |
| mmu05167 | Kaposi's sarcoma-associated herpesvirus infection |
| mmu05134 | Legionellosis |
| mmu05140 | Leishmaniasis |
| mmu05144 | Malaria |
| mmu05206 | MicroRNAs in cancer |
| mmu04064 | NF-kappa B signaling pathway |
| mmu04621 | NOD-like receptor signaling pathway |
| mmu04380 | Osteoclast differentiation |
| mmu05133 | Pertussis |
| mmu04145 | Phagosome |
| mmu05205 | Proteoglycans in cancer |
| mmu05323 | Rheumatoid arthritis |
| mmu05132 | Salmonella infection |
| mmu05150 | Staphylococcus aureus infection |
| mmu04350 | TGF-beta signaling pathway |
| mmu04668 | TNF signaling pathway |
| mmu04620 | Toll-like receptor signaling pathway |
| mmu05202 | Transcriptional misregulation in cancer |
| mmu05152 | Tuberculosis |

All pathways were also significant in the top 1000 sets of *Gm5483*, *Ccl4*, and *Il-1β*.

*Cxcl5* was located near the lower vertex of ΔAg (D region in Fig. 6), and its gene sets contained genes that turned to downregulated at the 24 h point (S2 Table B). Many GO terms associated with cytokines and wound healing were significant in the "BP" category of the *Cxcl5* set, as in the case of 3- and 5-fold upregulated sets (S2 Table C). Also, GO terms associated with cell membranes in the "CC" category and receptor binding and the activity of cytokines in the "MF" category were predominant. In addition, pathway analysis detected many pathways associated with cytokines and inflammation, such as mmu04060, which was significant in the 3- and 5-fold upregulated sets (Fig. S1 and S2 Table D). Moreover, infection pathways such as mmu05144 (Malaria) were also significant.

**S2 Table B. The number of genes with each fluctuation pattern in the top 100 to 1000 gene sets whose distance was close to the row score of Cxcl5 on the CA plot.**

| 6, 12, 24h | 100 set | 300 set | 500 set | 1000 set |
| --- | --- | --- | --- | --- |
| U, U, - | 46 | 127 | 197 | 356 |
| U, U, D | 1 | 3 | 7 | 16 |
| U, -, - | 0 | 2 | 10 | 33 |
| -, U, - | 0 | 4 | 15 | 59 |
| -, -, D | 0 | 0 | 0 | 14 |
| U, -, D | 0 | 0 | 0 | 2 |
| -, U, D | 0 | 0 | 0 | 4 |
| U, U, U | 53 | 163 | 253 | 421 |
| -, -, - | 0 | 1 | 18 | 95 |

"U: significantly upregulated, D: significantly downregulated, -: not fluctuated, at each time point (6, 12, 24 h post-injury in order). "

**S2 Table C. The number of genes belonging to specific GO terms in each set in the GO analysis of the top 100 and 1000 gene sets whose distances were close to each query gene on the CA plot.**

| GO ID | GO term | Cxcl5 top100 | Cxcl5 top1000 | Slpi top1000 | Saa3 top1000 | Slc26a10 top1000 | Myh7 top1000 | Tmem233 top1000 | Arg1 top1000 | Ly6f top1000 | Among all  55527 genes |
| --- | --- | --- | --- | --- | --- | --- | --- | --- | --- | --- | --- |
| GO:0006915 | Apoptotic process | **21** | **128** | **115** | **112** | 76 | 20 | 55 | **115** | 21 | 2652 |
| GO:0016477 | Cell migration | **21** | **124** | 70 | **84** | 53 | 16 | 28 | **91** | 18 | 1910 |
| GO:0006935 | Chemotaxis | **13** | **79** | 31 | **37** | 16 | 5 | 7 | **52** | 9 | 762 |
| GO:0043292 | Contractile fiber | 1 | 9 | 7 | 6 | 8 | 4 | 0 | 8 | 1 | 367 |
| GO:0005125 | Cytokine activity | **14** | **32** | 5 | 9 | 10 | 5 | 3 | 12 | 3 | 266 |
| GO:0001816 | Cytokine production | **21** | **85** | 41 | **47** | 17 | 7 | 11 | **68** | 6 | 950 |
| GO:0005737 | Cytoplasm | 29 | 354 | **507** | **535** | **449** | 109 | **370** | **511** | 156 | 15519 |
| GO:0006954 | Inflammatory response | **22** | **108** | 29 | **45** | 16 | 11 | 12 | **66** | 14 | 851 |
| GO:0005739 | Mitochondrion | 2 | 28 | 64 | 83 | 59 | 17 | **152** | **66** | 22 | 2584 |
| GO:0042692 | Muscle cell differentiation | 5 | 23 | 15 | 15 | 5 | 2 | 18 | 11 | 5 | 576 |
| GO:0006936 | Muscle contraction | 4 | 18 | 11 | 14 | 10 | 5 | 15 | 11 | 4 | 435 |
| GO:0061061 | Muscle structure development | **9** | **46** | 24 | 21 | 21 | 7 | 29 | 15 | 9 | 1001 |
| GO:0045445 | Myoblast differentiation | **3** | **13** | 3 | 1 | 3 | 1 | 2 | 1 | 2 | 133 |
| GO:0007520 | Myoblast fusion | **2** | **7** | 5 | 4 | 1 | 0 | 1 | 1 | 0 | 69 |
| GO:0014839 | Myoblast migration involved in  skeletal muscle regeneration | 0 | 0 | 0 | 0 | 0 | 0 | 0 | 0 | 0 | 4 |
| GO:1901265 | Nucleoside phosphate binding | 7 | 70 | **108** | **115** | 93 | 28 | 75 | **126** | 18 | 3057 |
| GO:0044459 | Plasma membrane part | 14 | **128** | **122** | **139** | 69 | 57 | 47 | **153** | 45 | 3642 |
| GO:0005102 | Receptor binding | **21** | **128** | 69 | 77 | 58 | 27 | 29 | 90 | 28 | 2253 |
| GO:0043269 | Regulation of ion transport | 8 | **44** | 41 | 35 | 21 | 12 | 21 | 42 | 10 | 989 |
| GO:0035914 | Skeletal muscle cell differentiation | **4** | **15** | 2 | 2 | 3 | 1 | 4 | 1 | 2 | 114 |
| GO:0003009 | Skeletal muscle contraction | 0 | 1 | 0 | 0 | 0 | 1 | **6** | 1 | 1 | 60 |
| GO:0006810 | Transport | **28** | **190** | **229** | **255** | 173 | 62 | 160 | **234** | 67 | 6431 |
| GO:0042060 | Wound healing | 4 | **50** | 26 | **28** | 19 | 4 | 9 | 26 | 1 | 555 |

Boldface letters indicate that the particular GO terms are significant at each time point.

**S2 Table D. Genes in the top 1000 sets of Cxcl5 included in the mmu4060 pathway and their FC values at each time point.**

| Probe name | Symbol | FC6 | FC12 | FC24 |
| --- | --- | --- | --- | --- |
| A_51_P317640 | Tgfb2 | 1.764 | 1.578 | 0.237 |
| A_65_P10913 | Tgfb2 | 2.261 | 2.505 | 0.316 |
| A_51_P463003 | Tslp | 1.297 | 1.743 | 0.388 |
| A_52_P586944 | Bmpr1b | 2.500 | 1.575 | 0.420 |
| A_51_P439299 | Relt | 1.770 | 2.279 | 0.531 |
| A_55_P2138386 | Il5 | 2.607 | 2.158 | 0.677 |
| A_55_P2007470 | Pdgfa | 2.291 | 3.236 | 0.738 |
| A_55_P2125588 | Pdgfa | 2.300 | 3.169 | 0.751 |
| A_55_P2075553 | Tnfsf18 | 1.529 | 3.706 | 0.759 |
| A_51_P331752 | Ccl11 | 3.021 | 3.187 | 0.765 |
| A_51_P239750 | Inhba | 7.251 | 5.187 | 0.869 |
| A_55_P2041422 | Il1rap | 4.810 | 5.459 | 0.872 |
| A_66_P136389 | Bmpr1b | 5.629 | 1.886 | 1.250 |
| A_51_P171075 | Csf2 | 28.974 | 8.177 | 1.382 |
| A_55_P2103249 | Cxcr1 | 12.029 | 22.805 | 1.419 |
| A_52_P106620 | Tnfrsf11b | 4.069 | 9.028 | 1.425 |
| A_52_P616356 | Ccr1 | 8.454 | 8.537 | 1.444 |
| A_51_P317176 | Csf3 | 44.432 | 24.269 | 1.938 |
| A_66_P123635 | Csf2rb | 7.192 | 8.073 | 2.140 |
| A_55_P2091676 | Fas | 4.400 | 5.172 | 2.285 |
| A_51_P271503 | Il1r1 | 8.155 | 9.644 | 2.309 |
| A_51_P195506 | Csf1 | 7.576 | 8.204 | 2.424 |
| A_51_P131408 | Tnfrsf12a | 8.832 | 14.530 | 2.554 |
| A_51_P322640 | Ccl24 | 4.575 | 9.283 | 2.753 |
| A_52_P467232 | Il1rap | 14.742 | 18.535 | 2.757 |
| A_51_P291361 | Osm | 48.380 | 28.617 | 3.005 |
| A_52_P517098 | Il18rap | 37.161 | 37.838 | 3.193 |
| A_51_P385099 | Tnf | 7.053 | 22.667 | 3.249 |
| A_55_P2034663 | Csf2rb2 | 10.691 | 16.946 | 3.266 |
| A_51_P267783 | Il11 | 10.807 | 7.373 | 3.385 |
| A_51_P319460 | Osmr | 7.749 | 9.269 | 3.768 |
| A_51_P146753 | Csf2rb2 | 9.151 | 10.556 | 4.010 |
| A_55_P2096422 | Inhbb | 11.752 | 10.744 | 4.023 |
| A_51_P235984 | Il10ra | 11.009 | 11.367 | 4.475 |
| A_52_P552194 | Il13ra1 | 15.592 | 21.706 | 6.358 |
| A_52_P52618 | Csf2rb | 25.604 | 31.170 | 6.619 |
| A_51_P348280 | Il17ra | 14.875 | 19.432 | 8.062 |
| A_51_P341465 | Csf2ra | 17.839 | 23.206 | 8.787 |
| A_51_P470079 | Il1r2 | 49.538 | 87.337 | 10.080 |
| A_55_P2004786 | Ccr1l1 | 57.216 | 22.191 | 10.413 |
| A_51_P114462 | Ccl17 | 34.496 | 28.590 | 11.816 |
| A_52_P559975 | Cxcr2 | 110.463 | 184.079 | 19.795 |
| A_51_P217463 | Cxcl2 | 467.168 | 300.084 | 22.094 |
| A_51_P208240 | Tnfsf14 | 64.623 | 145.738 | 22.698 |
| A_55_P1997756 | Il6 | 1049.196 | 256.990 | 29.908 |
| A_51_P363187 | Cxcl1 | 796.442 | 282.205 | 35.977 |
| A_51_P140710 | Ccl3 | 593.162 | 295.339 | 41.664 |
| A_51_P212782 | Il1b | 2140.053 | 1207.213 | 73.427 |
| A_51_P509573 | Ccl4 | 2871.902 | 1409.222 | 115.613 |
| A_51_P286737 | Ccl2 | 368.534 | 424.537 | 120.416 |
| A_51_P436652 | Ccl7 | 269.216 | 563.887 | 129.603 |
| A_55_P1990032 | Cxcl5 | 3163.201 | 2260.397 | 227.743 |

Apart from the query genes, *Ly6f* and *Arg1* genes that were located at the neighborhood of the left and right vertex of ΔAg, respectively, were also analyzed in the same manner (Fig. 7d). Although there was a smaller number of significant GO terms and pathways in the top 1,000 set of *Arg1* compared to *Cxcl5*, they were associated with cytokines, leukocytes, and lymphocytes as in the case of the top 1,000 set of *Cxcl5*. Only one GO term and no pathways were significant in the top 100 set of the *Ly6f*, and only two genes were annotated with a GO term.

The pathways that were significant in the top 1,000 set of the downregulated query genes at the 24-h point are shown in S2 Table E. The pathway of mmu00190 (Oxidative phosphorylation) was significant in these sets (except for Gm6288). Oxidative phosphorylation for ATP synthesis is a mitochondrial function, and mitochondria can be affected by sepsis [1]. The GO term associated with mitochondria was significant only in the set of *Tmem233*. There were a small number of genes that were downregulated by more than 3-fold in mmu00190, so those GO terms were not significant in the more than 3 and 5-fold downregulated sets. Pathways of neurological disease groups such as Alzheimer's disease (mmu05010) associated with mitochondrial dysfunction were also significant.

**S2 Table E. Pathways that were significant in the pathway analysis of the top 1000 set of query genes downregulated at the 24 h time point.**

| Entry | Name |
| --- | --- |
| Plcd4 |  |
| mmu05010 | Alzheimer's disease |
| mmu01200 | Carbon metabolism |
| mmu04260 | Cardiac muscle contraction |
| mmu00020 | Citrate cycle (TCA cycle) |
| mmu04066 | HIF-1 signaling pathway |
| mmu05016 | Huntington's disease |
| mmu01100 | Metabolic pathways |
| mmu04932 | Non-alcoholic fatty liver disease (NAFLD) |
| mmu00190 | Oxidative phosphorylation |
| mmu05012 | Parkinson's disease |
| mmu00620 | Pyruvate metabolism |
| mmu04723 | Retrograde endocannabinoid signaling |
|  |  |
| Ostn |  |
| mmu04261 | Adrenergic signaling in cardiomyocytes |
| mmu05010 | Alzheimer's disease |
| mmu01230 | Biosynthesis of amino acids |
| mmu01200 | Carbon metabolism |
| mmu04260 | Cardiac muscle contraction |
| mmu05414 | Dilated cardiomyopathy (DCM) |
| mmu00010 | Glycolysis / Gluconeogenesis |
| mmu05016 | Huntington's disease |
| mmu05410 | Hypertrophic cardiomyopathy (HCM) |
| mmu04010 | MAPK signaling pathway |
| mmu01100 | Metabolic pathways |
| mmu04932 | Non-alcoholic fatty liver disease (NAFLD) |
| mmu00190 | Oxidative phosphorylation |
| mmu04921 | Oxytocin signaling pathway |
| mmu05012 | Parkinson's disease |
|  |  |
| Mettl11b |  |
| mmu05010 | Alzheimer's disease |
| mmu01200 | Carbon metabolism |
| mmu04260 | Cardiac muscle contraction |
| mmu00020 | Citrate cycle (TCA cycle) |
| mmu05016 | Huntington's disease |
| mmu01100 | Metabolic pathways |
| mmu04932 | Non-alcoholic fatty liver disease (NAFLD) |
| mmu00190 | Oxidative phosphorylation |
| mmu05012 | Parkinson's disease |
| mmu04723 | Retrograde endocannabinoid signaling |
|  |  |
| Gm6288 |  |
| mmu04740 | Olfactory transduction |
|  |  |
| Tmem233 |  |
| mmu05010 | Alzheimer's disease |
| mmu01200 | Carbon metabolism |
| mmu00020 | Citrate cycle (TCA cycle) |
| mmu05016 | Huntington's disease |
| mmu01100 | Metabolic pathways |
| mmu04932 | Non-alcoholic fatty liver disease (NAFLD) |
| mmu00190 | Oxidative phosphorylation |
| mmu05012 | Parkinson's disease |
| mmu04723 | Retrograde endocannabinoid signaling |

Reference

[1] Brealey D, Brand M, Hargreaves I, Heales S, Land J, Smolenski R, et al. Association between mitochondrial dysfunction and severity and outcome of septic shock. Lancet. 2002 Jul 20;360(9328):219-23.
