# Supplementary material for: Time course analysis of large-scale gene expression in incised muscle using correspondence analysis
Source: PLoS One. 2020 Mar 25;15(3):e0230737. doi: 10.1371/journal.pone.0230737 (PMC7094855; doi:10.1371/journal.pone.0230737)
Supplement: Attachment — Submitted filename: Response to Reviewers.docx [file pone.0230737.s014.docx]

We thank the reviewers for their advice and suggestions. We have taken all comments into consideration and have rewritten the manuscript thoroughly. Our response to each comment and the revisions made in the manuscript are described below.

Reviewer #1

1. The discussion should be revise using the result obtained and should not only be based on literature.

**Response**

Thank you for your suggestion. We compared some of the references in the manuscript with the results of this study and added some discussion.

**The following descriptions were added to the Discussion, respectively.**

“Discussion”, p30-31, L484-487,

The large FC values of these genes in our microarray results were considered to be consistent with their bioactivity after injury (except for *Gm5483*, which is less well studied than other cytokines).

“Discussion”, p33, L524-526,

Considering that its FC value was maintained more than 100 even at 24 h after injury, *Gm5483* was seemed to play a certain role in both the inflammatory response of early phase and the muscle repair of late phase after injury.

“Discussion”, p35, L558-560,

*Cd72*, a negative regulator of B-cell responsiveness, would have been downregulated until 12 h to promote the inflammatory response, and then turned to upregulation at 24 h to inhibit the excessive inflammatory response.

2. The author should add the references in the bibliography: 1.shen et al 2019 Front Physiol 10 (1298)

**The suggested literature was added to the References.**

“References”, p44, L706-708,

[20] Shen Y, Zhang R, Xu L, Wan Q, Zhu J, Gu J, et al. Microarray Analysis of Gene Expression Provides New Insights Into Denervation-Induced Skeletal Muscle Atrophy. Front Physiol. 2019 Oct 11;10:1298. doi: 10.3389/fphys.2019.01298. eCollection 2019.

**Also, we have added the following description to the Discussion.**

“Discussion”, p30, L468-470,

Shen et al. [20] performed PCA to visualize time-dependent expression pattern of microarray data of tibialis anterior muscle after peripheral nerve injury in rats.
